# Antimicrobial usage in broiler chicken production in the United States, 2013–2021

**DOI:** 10.3389/fvets.2023.1139908

**Published:** 2023-04-17

**Authors:** Randall S. Singer, Nora F. D. Schrag, Isabel Ricke, Michael D. Apley

**Affiliations:** ^1^Department of Veterinary and Biomedical Sciences, University of Minnesota, Saint Paul, MN, United States; ^2^Mindwalk Consulting Group, LLC, Falcon Heights, MN, United States; ^3^Livestock Veterinary Resources, LLC, Manhattan, KS, United States; ^4^Department of Clinical Sciences, College of Veterinary Medicine, Kansas State University, Manhattan, KS, United States

**Keywords:** broiler chickens, antimicrobial use, antimicrobial stewardship, epidemiological monitoring, necrotic enteritis, gangrenous dermatitis, colibacillosis

## Abstract

Although efforts to improve antimicrobial stewardship should include the collection of antimicrobial use data, most antimicrobial datasets collected at the national level consist of antimicrobial sales data which cannot inform stewardship. These data lack context, such as information regarding target species, disease indication, and regimen specifics like dose, route and duration. Therefore, the goal of this study was to develop a system for collecting data on the use of antimicrobials in the U.S. broiler chicken industry. This study utilized a public-private partnership to enable collection and protection of sensitive data from an extremely large industry while releasing deidentified and aggregated information regarding the details of antimicrobial use on U.S. broiler chicken farms over time. Participation was voluntary. Data were collected for the period 2013 through 2021 and are reported on a calendar year basis. Using production statistics from USDA:NASS as a denominator, the data supplied by participating companies represented approximately 82.1% of broiler chicken production in the U.S. in 2013, approximately 88.6% in 2017, and approximately 85.0% in 2021. The data that were submitted for 2021 are based on approximately 7,826,121,178 chickens slaughtered and 50,550,817,859 pounds liveweight produced. Granular flock-level treatment records were available for 75–90% of the birds represented in the 2018–2021 dataset. There was no use of antimicrobials in the hatchery for the years 2020 and 2021. Medically important in-feed antimicrobial use decreased substantially, with all in-feed tetracycline use being eliminated by 2020, and the use of virginiamycin being reduced by more than 97% since 2013. Medically important water-soluble antimicrobials are used for the treatment of disease in broiler production. Use decreased substantially for most water-soluble antimicrobials. The most important diseases necessitating treatment were necrotic enteritis and gangrenous dermatitis as well as *E. coli*-related disease. A focus on reducing the incidence of these diseases would reduce the need for antimicrobial therapy but will require an investment in research to find efficacious and cost-effective interventions for these diseases.

## 1. Introduction

Making improvements in antimicrobial stewardship (AMS) is critical for ensuring the effectiveness of antimicrobials that are available for use in human and veterinary medicine (1–3). According to the American Veterinary Medical Association (AVMA), AMS is defined as “the actions veterinarians take individually and as a profession to preserve the effectiveness and availability of antimicrobial drugs through conscientious oversight and responsible medical decision-making while safeguarding animal, public, and environmental health” (2, 3). AMS programs in animal agriculture must include systems for collecting on-farm antimicrobial use (AMU) data that include information regarding the principal indications for AMU and details about each administration (dose, route, duration, age of animals at onset of treatment) of specific antimicrobial compounds.

Antimicrobial sales volumes are the most common form of data currently being collected globally, in part because on-farm AMU datasets that are representative of national production are much more difficult to collect. Antimicrobial sales data lack context for how the antimicrobials are actually being used; specifically, they provide no information regarding the intended reason for use and typically have no information about route or duration of administration. Although it has been suggested that national antimicrobial sales data can be used to set policy regarding national AMS (4), for example by using sales data collected and reported by the European Surveillance of Veterinary Antimicrobial Consumption (ESVAC) (5), these data do not provide information regarding the incidence of disease in specific herds or flocks nor the intended use of the antimicrobials included in the sales data. It is therefore unclear how national antimicrobial sales data alone are useful in assisting with AMS activities. The data reported here are intended to provide the granularity necessary for evaluation of the patterns and trends of AMU in broiler chicken production.

The U.S. Food and Drug Administration (FDA) has made changes to antimicrobial policy in recent years, and we have previously described some of the changes in our previous publication of AMU in broilers (6). Some of the key changes that resulted from these policies included: medically important antimicrobials in food-producing animals are no longer available for growth promotion/feed efficiency purposes and medically important antimicrobials administered in the feed or water of food-producing animals must occur with veterinary oversight, in the form of a Veterinary Feed Directive (VFD) (for feed-administered medically important antimicrobials) or prescription (for medically important antimicrobials administered in water) (7–9). FDA's Guidance for Industry (GFI) #152 (10) defines “medically important” antimicrobials (i.e., importance to human medical therapy), and Appendix A of that document provides a list and ranking of antimicrobials considered medically important in the U.S.; this list serves as the operating classification system for the data presented in this U.S.-based effort.

Similar to other countries, the main national dataset that exists in the U.S. is the antimicrobial sales data that are collected by the FDA (11). However, the FDA acknowledges in the sales data reports that “the data are not intended to be a substitute for actual usage data and should be used in conjunction with on-farm species-specific data on antimicrobial use (11).” In 2020, we published data regarding on-farm use of antimicrobials in U.S. broiler chicken production over the period 2013 through 2017 (6). This national effort represented between 80 and 90% of the annual broiler chicken production in the U.S, with more than 7,400,000,000 slaughtered chickens and more than 47 billion pounds liveweight represented in the 2017 data. That first effort focused on the total amounts of different antimicrobials used on-farm, with each antimicrobial class stratified by route of administration (hatchery, feed and water). For the 2017 data, we presented the targeted diseases for which the water-soluble antimicrobials were used. Our goal, though, was to collect more granular data from the U.S. broiler production system so that more details could be provided regarding the diseases being treated, age at onset of disease requiring therapy, duration of therapy, and the number of prescriptions during each calendar year. Therefore, the objective of this current effort was to collect granular on-farm antimicrobial use data from the U.S. broiler chicken industry and to have it be representative of the national flock.

## 2. Materials and methods

### 2.1. Enrollment

The objective of this initiative was to recruit commercial broiler chicken companies in the United States that represent a large fraction of annual production. As per the USDA National Agricultural Statistics Service (NASS) estimates, about 9,210,889,000 broilers were slaughtered in 2021 (12). Details about the enrollment process are presented in our previous publication (6). Most of the enrolled companies had already participated in the previous data collection effort. Companies were informed that they would need to provide information on production parameters, hatchery antimicrobial use and feed and water antimicrobial use, spanning the years 2018 to 2021. Data from 2013 to 2017 had already been provided in the previous effort, although some companies amended some of their previous records. Specifically, some companies were able to provide granular flock-level records for these earlier years. Participation was voluntary, and all companies were assured that their data would be kept confidential and that only aggregated industry data would be made public.

### 2.2. Data collection, aggregation and reporting

The information gathered from participating broiler companies was combined and summarized as yearly totals. There was no single, standardized format to the data submitted by participating companies. Consequently, data transformation, aggregation, and analysis into estimates of AMU was not a simple process. Details about the data collection process are presented in our previous publication (6). Briefly, some data were submitted as prescription records from individual flocks. Some data were provided as total amounts of each antimicrobial drug that was used during the calendar year. These data were often stratified by disease indication for each antimicrobial and are similar to antimicrobial sales data in that they lack the granular details of AMU; unlike national antimicrobial sales datasets, however, the disease indication stratification provided some context.

For data on water-soluble antimicrobials, some companies only submitted the number of birds treated with each antimicrobial for different illnesses but did not provide the total amount of antimicrobial administered to each affected flock. In these cases, a table of water consumption provided by the company was used to calculate the amount of antimicrobial that would have been given, based on the age of the birds, the dose of antimicrobial administered, and the duration of treatment. To demonstrate this calculation, we use an example of a prototypical flock of 35,000 broilers that is to receive treatment with tylosin for necrotic enteritis. In this example, assume that the birds are 20 days old and receive treatment for 3 days. Tylosin administered in the water comes in packs of 50 grams of active substance per pack. When mixed according to label, the final concentration will be 0.1032 grams/liter of drinking water (the label in the U.S. is 50 grams/128 gallons of drinking water). Based on the water consumption table, birds at this age drink an estimated 566 liters of water per 1,000 birds over 3 days. The total grams of tylosin estimated to be administered in the water over the 3-day course of therapy is: 35,000 birds × 566 liters/1,000 birds × 0.1032 grams/liter = 2,044.4 grams. The veterinarian would then send 41 packs of tylosin to the farm, totaling 2,050 grams. For this analysis, we used the rounded up number of packs, calculated as above.

Details regarding data validation and aggregation are presented in our previous publication (6). After validation, data from each company were imported into R 4.2.2 (13) and aggregated. All analyses and graphing of the AMU records were performed in R.

The annual estimates for antimicrobial use are presented by active substance within each class and are divided into two categories: medically important (MI) and not medically important (NMI), based on FDA classifications (10). These classifications for broilers are shown in Supplementary Tables 1, 2. The AMU data in this report are not combined across classes or methods of administration because dose and potency/molecular weight of antimicrobial substances can vary greatly (11).

For AMU totals, we report the estimates in a similar fashion to our previous publication (6). Briefly, antimicrobials used in the hatchery are reported as totals and as mg per 100 birds placed. The in-feed and water-soluble administration data are reported as totals and as mg/kg liveweight slaughtered. All AMU totals that were collected during this study are included in the Supplementary material.

### 2.3. Granular antimicrobial use data analysis

Flock-level treatment records were submitted by many of the companies regarding their use of water-soluble antimicrobials during the period 2018 through 2021. Annual data representing 75–90% of the broilers in the study had this level of granularity. The full prescription records included the disease being treated, the number of animals being treated, the age of the birds at the start of therapy, the duration of therapy, and the amount of antimicrobial sent to the farm for the prescription. We refer to these records as prescriptions instead of treatments because the records that we received were the actual prescriptions written by the veterinarian and were not records of actual on-farm treatment.

Several analyses were performed with the flock prescription records. First, we report the number of birds prescribed water-soluble treatment per 1,000 birds slaughtered by year and by disease indication. Second, we report the relative frequency of birds beginning treatment for each disease indication by age at the start of therapy for some of the most commonly treated diseases. Third, we report the number of birds prescribed each class of water-soluble treatment per 1,000 birds slaughtered by year and by disease indication. Fourth, we report the relative frequency of prescription durations for each disease indication. Finally, we report the percentage of birds prescribed treatment with a given antimicrobial class and the total grams of each water-soluble antimicrobial class administered for each disease indication.

## 3. Results

### 3.1. Enrollment

The companies that submitted data for this project produced the majority of broiler chickens in the U.S. during each year of the study. The companies that participated encompassed all types of production, including conventional, raised without antimicrobials (RWA) and organic. Most companies raised animals in multiple types of production systems; the data that were submitted by participating companies cannot be stratified by production type.

The 2013 dataset included 7,099,320,561 chicks placed, 6,825,820,035 broilers slaughtered and 41,330,539,038 pounds (lbs) liveweight produced in 2013 (Table 1). The 2017 dataset included 7,980,575,828 chicks placed, 7,609,645,310 broilers slaughtered and 49,008,158,712 lbs liveweight produced (Table 1). The 2021 dataset included 8,245,081,339 chicks placed, 7,826,121,178 broilers slaughtered and 50,550,817,859 lbs liveweight produced (Table 1). Data for administration of water-soluble antimicrobials were more difficult to obtain for some of the companies in 2013 to 2014, and thus the total number of broilers covered in the dataset for the water-soluble administration is less than for the hatchery, ionophore and in-feed administrations in these years, as previously reported (6). The dataset accounts for approximately 80.3 and 82.1% of the broilers slaughtered and total pounds liveweight, respectively, in the U.S. in 2013, as reported by USDA:NASS. These figures increased to approximately 85.0% in 2021. There was a drop in participation from 2017 to 2019 followed by an increase from 2019 to 2021 due to a change in companies voluntarily participating. All denominator data collected during this study are included in the Supplementary material.

**Table 1:** Broiler chicken production data included in the antimicrobial datasets submitted by participating companies for each year of the study.

	**2013**	**2014**	**2015**	**2016**	**2017**
**Hatchery antimicrobial denominators**					
Chicks placed	7,099,320,561	6,989,549,333	7,652,063,534	7,799,081,679	7,980,575,828
**Study production denominators**					
Head slaughtered	6,825,820,035	6,688,329,433	7,294,241,950	7,454,249,403	7,609,645,310
Liveweight (lbs)	41,330,539,038	41,482,589,732	46,005,065,433	47,796,265,052	49,008,158,712
**USDA:NASS statistics**					
Head slaughtered	8,503,750,000	8,525,393,000	8,688,462,000	8,768,399,000	8,916,083,000
Liveweight (lbs)	50,357,463,000	51,225,964,000	53,169,030,000	54,036,929,000	55,314,913,000
**Percentage of U.S. broiler chicken production**					
Head slaughtered	80.3%	78.5%	84.0%	85.0%	85.3%
Liveweight (lbs)	82.1%	81.0%	86.5%	88.5%	88.6%
	**2018**	**2019**	**2020**	**2021**	
**Hatchery antimicrobial denominators**					
Chicks placed	7,743,582,472	7,935,362,665	8,284,444,848	8,245,081,339	
**Study production denominators**					
Head slaughtered	7,364,220,275	7,532,062,726	7,909,663,865	7,826,121,178	
Liveweight (lbs)	46,823,629,823	48,354,774,429	50,863,388,193	50,550,817,859	
**USDA:NASS statistics**					
Head slaughtered	9,034,504,000	9,224,243,000	9,229,801,000	9,210,889,000	
Liveweight (lbs)	56,541,518,000	58,286,997,000	59,155,652,000	59,486,734,000	
**Percentage of U.S. broiler chicken production**					
Head slaughtered	81.5%	81.7%	85.7%	85.0%	
Liveweight (lbs)	82.8%	83.0%	86.0%	85.0%	

Annual U.S. broiler production statistics are taken from USDA:NASS (12).

The percentage of annual U.S. broiler production calculation is based on the study production data (numerator) and USDA:NASS data (denominator).

### 3.2. Hatchery antimicrobials

**Figure 1:**
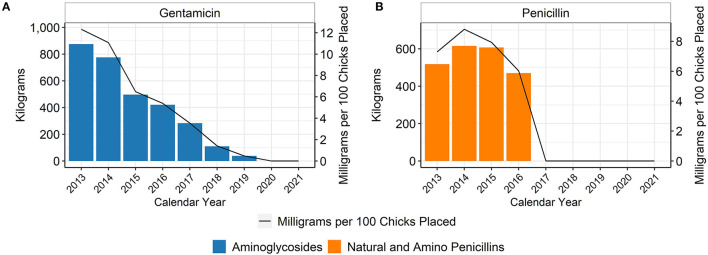
Gentamicin **(A)** and penicillin **(B)** used in broiler hatcheries, 2013–2021. Total kilograms are shown by the bars (left Y-axis) and total mg/100 birds placed are shown by the line (right Y-axis).

**Figure 2:**
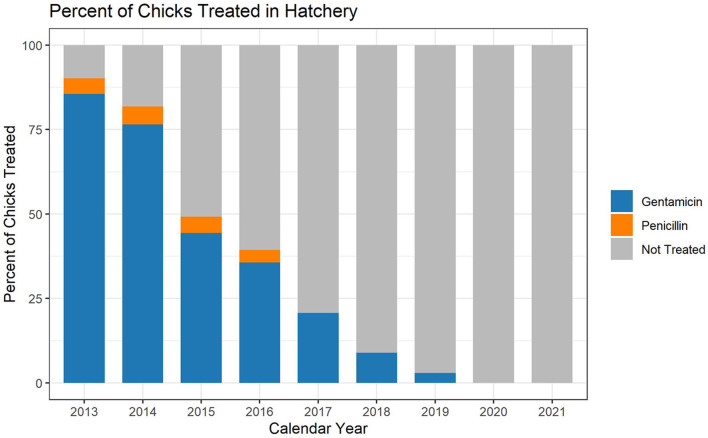
Broiler hatchery antimicrobial use during the years 2013–2021, as a percentage of total birds placed. The graph shows the percentage of birds placed that received gentamicin, penicillin or no antimicrobial.

The hatchery data are based on the annual placement of between 6,989,549,333 and 8,284,444,848 chicks, depending on the year (Table 1). In the broiler chicken industry, hatchery antimicrobials are generally given in ovo rather than subcutaneously to day-old chicks. When using the metric of mg of antimicrobial per 100 birds placed, hatchery use of gentamicin decreased every year between 2013 and 2019 (Figure 1A). Penicillin was not used in the hatcheries represented in this dataset after 2016 (Figure 1B). There was no reported use of ceftiofur in the broiler hatcheries during this study, as this study began 1 year after most extra-label uses of cephalosporins in livestock and poultry were prohibited in the U.S. (14). After 2019, there was no reported use of any antimicrobial in broiler hatcheries in this dataset, which represents more than 85% of production in the U.S. (Figure 2). The percentage of broiler chicks placed that received hatchery antimicrobials decreased from approximately 90% in 2013 to < 3% in 2019, and then was 0% in 2020 and 2021 (Figure 2).

### 3.3. In-feed antimicrobials

**Figure 3:**
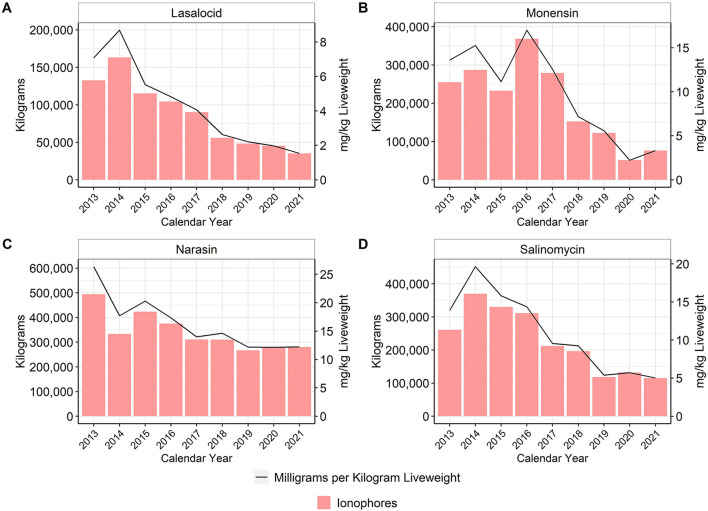
Lasalocid **(A)**, monensin **(B)**, narasin **(C)**, and salinomycin **(D)** used in broiler feed, 2013–2021. Total kilograms are shown by the bars (left Y-axis) and total mg/kg liveweight are shown by the line (right Y-axis).

In the U.S., ionophores are classified as NMI antimicrobial drugs, whereas they are considered coccidiostats in many countries, including the European Union (5). The U.S. broiler industry has four ionophores approved for use in production: lasalocid, narasin, monensin, and salinomycin (15). There was an approximate 95% reduction of lasalocid use, 75% reduction of monensin use, 53% reduction of narasin use, and 62% reduction of salinomycin use between 2013 and 2021 (Figure 3).

**Figure 4:**
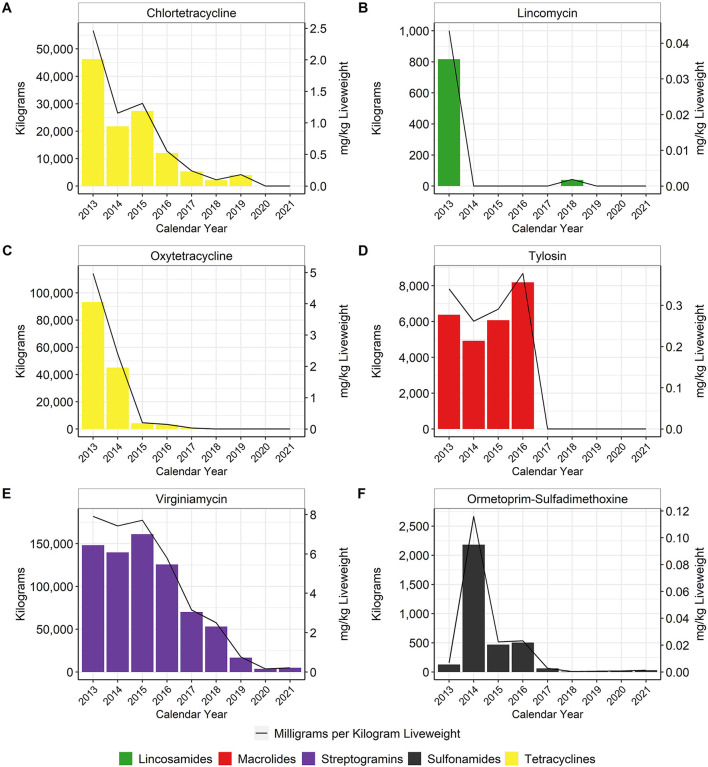
Medically Important antimicrobials chlortetracycline **(A)**, lincomycin **(B)**, oxytetracycline **(C)**, tylosin **(D)**, virginiamycin **(E)**, and ormetoprim-sulfadimethoxine **(F)** used in broiler feed, 2013–2021. Total kilograms are shown by the bars (left Y-axis) and total mg/kg liveweight are shown by the line (right Y-axis).

**Figure 5:**
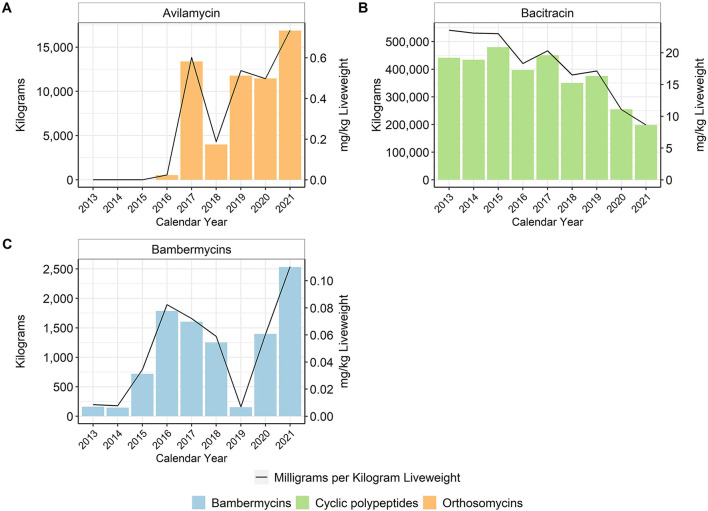
Not Medically Important antimicrobials avilamycin **(A)**, bacitracin **(B)**, and bambermycins **(C)** used in broiler feed, 2013–2021. Total kilograms are shown by the bars (left Y-axis) and total mg/kg liveweight are shown by the line (right Y-axis).

In general, in-feed antimicrobial use decreased over time, particularly for the MI antimicrobials. When using the metric of total mg/kg liveweight produced, there was an approximate 96% reduction of tetracycline use between 2013 and 2017, with no reported tetracycline use in-feed in 2020 or 2021 (Figure 4). Virginiamycin use decreased approximately 97% between 2013 and 2021. In-feed bacitracin, an NMI antimicrobial, was used primarily for the prevention of necrotic enteritis, although its use decreased by more than 65% between 2013 and 2021 (Figure 5). The use of avilamycin and bambermycins, both NMI antimicrobials increased in usage over time.

### 3.4. Water-soluble antimicrobials

**Figure 6:**
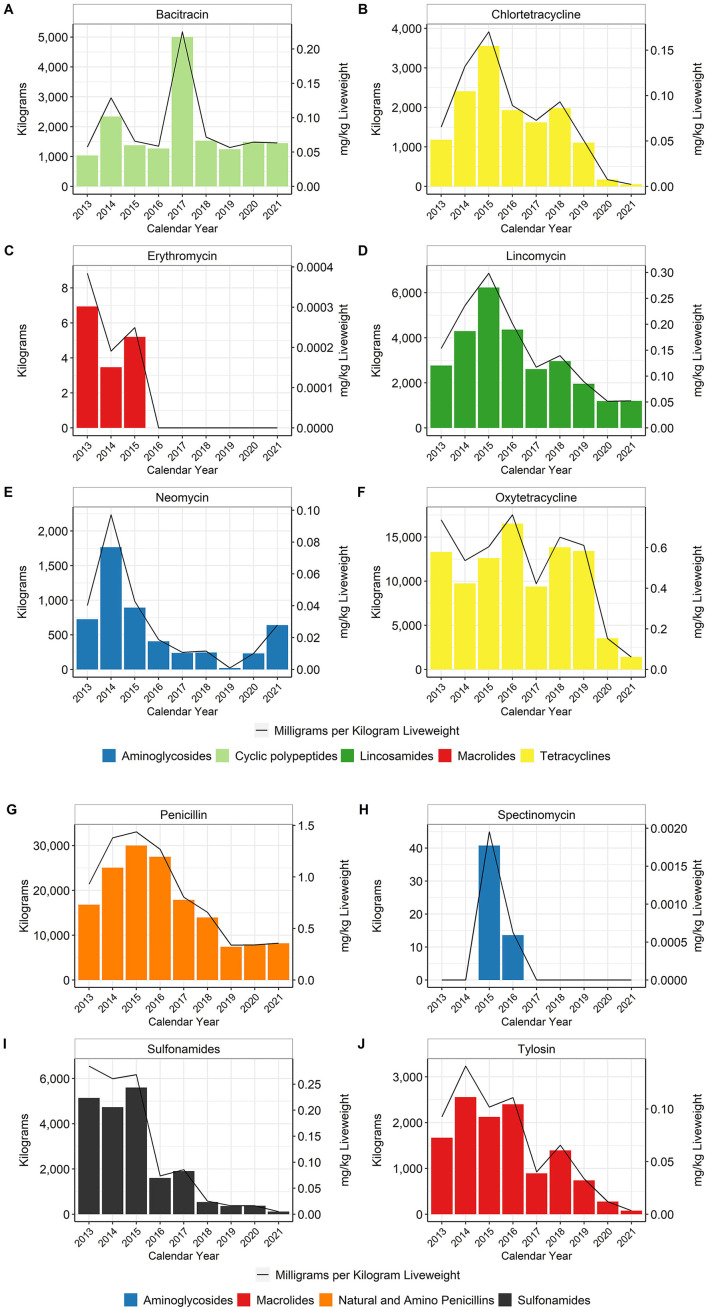
Water-soluble antimicrobials bacitracin **(A)**, chlortetracycline **(B)**, erythromycin **(C)**, lincomycin **(D)**, neomycin **(E)**, oxytetracycline **(F)**, penicillin **(G)**, spectinomycin **(H)**, sulfonamides **(I)**, and tylosin **(J)** used in broiler water, 2013–2021. Total kilograms are shown by the bars (left Y-axis) and total mg/kg liveweight are shown by the line (right Y-axis). Bacitracin is a Not Medically Important antimicrobial whereas the rest are considered Medically Important.

Data for the water-soluble antimicrobials are presented for each active substance except for the sulfonamide class, for which it can be difficult to separate the different active ingredients from the company records (Supplementary Table 1). Chlortetracycline and oxytetracycline were both used in water, although oxytetracycline was used more frequently than chlortetracycline. The data are reported for each individual substance as well as for the tetracycline class as a whole. Water-soluble administration remains the key manner for treatment of disease (Figure 6), as sick birds my consume less feed but will typically maintain water consumption (16).

When using the metric of total mg/kg liveweight produced, water-soluble penicillin use decreased approximately 62% between 2013 and 2021 (Figure 6G). Water-soluble lincomycin use decreased approximately 66% between 2013 and 2021 (Figure 6D). Usage of these two antimicrobials fluctuated between 2013 and 2017 but then declined steadily from 2018 to 2021. Water-soluble tetracycline use, as a class, decreased approximately 92% between 2013 and 2021 (Figures 6B, F). Water-soluble sulfonamide and tylosin use decreased approximately 98 and 95%, respectively, between 2013 and 2021 (Figures 6I, J). Use of bacitracin, the only water-soluble NMI antimicrobial with reported use during the study period, increased almost fourfold in 2017 and then returned to pre-2017 levels through 2021 (Figure 6A).

### 3.5. Granular antimicrobial use data analysis

**Figure 7:**
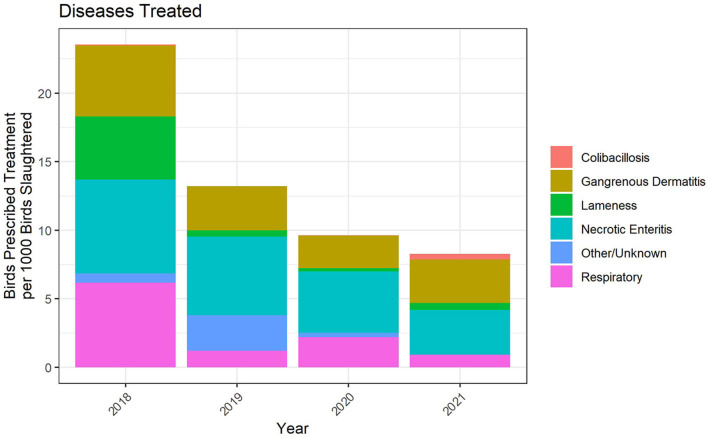
Disease indications treated with water-soluble antimicrobial administrations, 2018–2021. The figures depict the number of birds prescribed treatment for each disease indication per 1,000 birds slaughtered.

For the 2018 through 2021 data, flock-level treatment records for antimicrobials administered *via* the water represented between 75 and 90% of broiler chickens in the study. The indications for treatment were categorized into six disease classifications, one of which was Other/Unknown (Figure 7). There are no standardized disease classifications within the U.S. broiler chicken industry. We therefore categorized the recorded disease indications with input from participating veterinarians. There were instances of airsacculitis, likely caused by *E. coli*, that were included in the Respiratory category rather than the Colibacillosis category. In the future, we will work improve the disease classification categories and attempt to create a more standardized list across the industry for reporting purposes.

Based on the metric of birds prescribed treatment per 1,000 birds slaughtered, necrotic enteritis (NE), gangrenous dermatitis (GD) and respiratory disease were the three main disease classifications for which water-soluble antimicrobials were used. The number of birds prescribed treatment per 1,000 birds slaughtered decreased substantially over the period 2018–2021, to a level of ~8 prescriptions per 1,000 birds slaughtered in 2021 (Figure 7).

**Figure 8:**
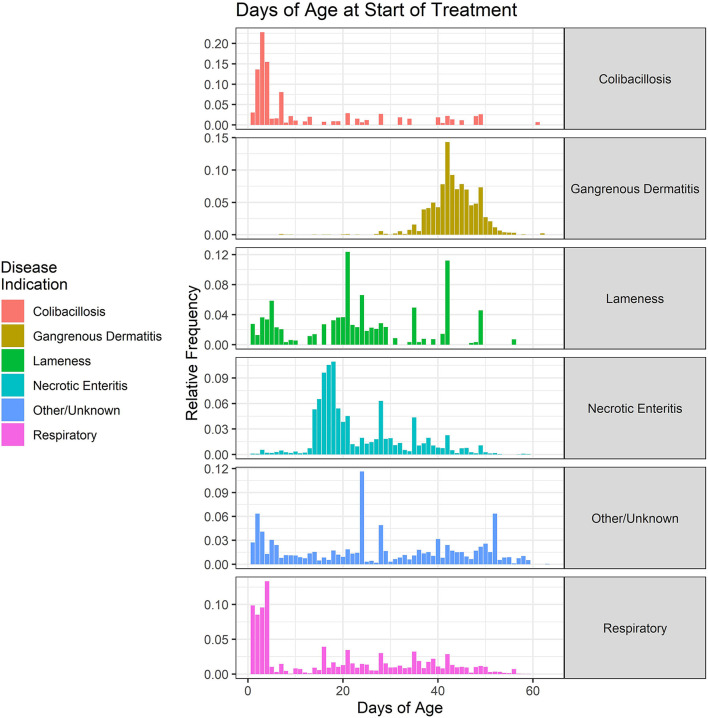
Age (in days) of the start of antimicrobial administrations in the water of broiler chickens by disease indication, 2018–2021. Each disease indication panel depicts the relative frequency of antimicrobial starting ages (in days) for the respective disease.

The ages of onset of treatment for the six disease classifications are shown in Figure 8. The data are collapsed over the 2018–2021 period. Colibacillosis and some respiratory diseases predominantly affect the young chicks, with most treatments beginning between 1 and 5 days of age. The colibacillosis disease category includes omphalitis, which only affects the newly hatched chicks. Necrotic enteritis tends to occur between 14 and 28 days of age, often related to the cycling of coccidial parasites in the broiler environment. Conversely, GD predominantly affects the older chickens, with most treatments beginning after 40 days of age. The respiratory disease category represents a number of different disease conditions, including airsacculitis, and has a wide distribution of onset ages.

**Figure 9:**
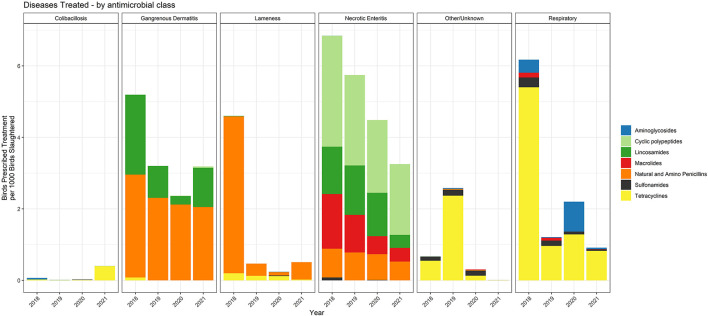
Number of water-soluble prescriptions for each antimicrobial class and disease indication, 2018–2021. The figures depict the number of birds prescribed treatment per 1,000 birds slaughtered.

The specific antimicrobial classes prescribed for each of the disease classifications over the 2018–2021 period are shown in Figure 9. The data are presented with the metric of birds prescribed treatment per 1,000 birds slaughtered. Necrotic enteritis and GD were the two disease classifications with the most prescriptions per 1,000 birds slaughtered. While penicillin and lincomycin were commonly used for treatment of GD, treatment of NE included bacitracin and tylosin in addition to penicillin and lincomycin. Illnesses caused by *E. coli*, as well as the majority of respiratory disease, were treated with tetracyclines.

**Figure 10:**
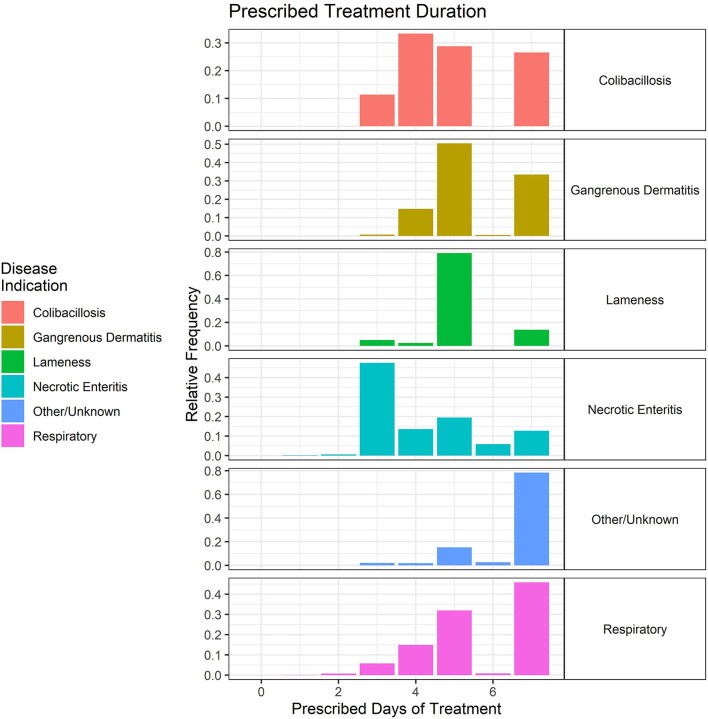
Duration (in days) of prescribed antimicrobial administrations in the water of broiler chickens by disease indication, 2018–2021. Each disease indication panel depicts the relative frequency of antimicrobial prescription durations (in days) for the respective disease.

The duration of treatment for these key disease classifications was variable but typically ranged from 3 to 7 days, regardless of disease (Figure 10). The data are shown as the relative frequency of prescription durations and are a composite of the treatment records from 2018 to 2021. Although the scale cuts off at 8 days, there were seven total prescriptions in the dataset that were for longer durations (10–14 days) for GD.

**Figure 11:**
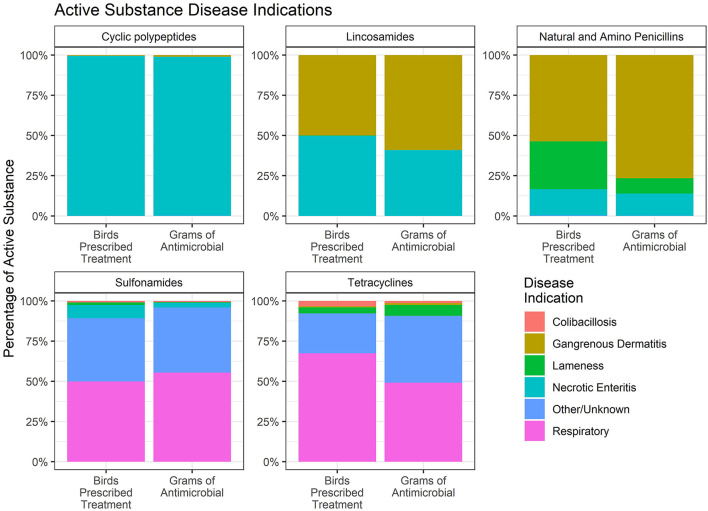
Percentage of antimicrobial use in the water of broiler chickens by disease indication, 2018–2021. Each figure only includes data for those birds that received the given antimicrobial. The figures depict the percentage of birds receiving therapy with the given antimicrobial (Birds Prescribed Treatment) and the percentage of total grams of the antimicrobial used by disease indication (Grams of Antimicrobial).

Finally, the percentage of each water-soluble antimicrobial that was administered for each disease indication was estimated. Data are presented as the percentage of birds prescribed treatment and the total grams of each antimicrobial administered for each disease indication (Figure 11). Penicillin and lincomycin were both used for the treatment of NE and GD. The figure shows that the percentage of birds receiving lincomycin for these two diseases was about equal, whereas more GD-affected birds were treated with penicillin than lincomycin. The amount of penicillin and lincomycin, by weight, was greater for GD than for NE. The disparity between the two measures is due to the fact that GD affects older birds than NE, and therefore, the proportion of use measured by weight (grams) is greater than the proportion of use measured by number of birds for GD.

## 4. Discussion

The data in this study represent the second phase of a large, industrywide effort to capture antimicrobial use information from the broiler chicken industry of the U.S. Participation rates were high for this 2013 to 2021 effort, with approximately 85.0% of 2021 U.S. broiler chicken production represented in the analysis, based on liveweight data reported by USDA:NASS (Table 1).

In general, there were substantial reductions in AMU in the largest U.S. broiler production companies between 2013 and 2021. Possible explanations for these reductions include veterinary oversight of all medically important antimicrobial administrations in the feed or water since January 2017 (7, 8), overall improved AMS, increased production of birds raised without antimicrobials, focus on improved preventive medicine and animal husbandry practices to reduce the need for antimicrobials, shifts to the use of non-medically important antimicrobials, and early diagnosis and intervention with non-antimicrobial therapies. With respect to AMS, there were noticeable improvements in data management over the nine-year time span of this report. We were able to obtain granular treatment records from 75 to 90% of the broiler chickens in the 2018–2021 dataset. This allowed us to begin providing more detail regarding the manner in which the antimicrobials are being administered. The percentage of broiler chicken production being raised without antimicrobials is currently estimated to be between 50 and 60% and has been relatively static for the past few years (17). Veterinarians seemed to be switching from MI to NMI antimicrobials, when possible, as documented in the patterns of usage for the in-feed and water-soluble antimicrobials (Figure 4). Other external factors may have also influenced the patterns of use of specific antimicrobials in this dataset. For example, there has been a documented shortage of penicillin, beginning during the COVID pandemic (18), although the impact of this shortage appeared to affect U.S. turkey companies more than broiler chickens.

There were other possible limitations to this effort to collect antimicrobial use data from U.S. broiler chicken production. First, there was some turnover in composition of companies that chose to voluntarily participate in this project. Because antimicrobial decisions can vary by company and by veterinarian within a company, variation in antimicrobial usage patterns can be affected by the companies that participate in a given year and does not necessarily reflect changes in disease incidence or AMS. Second, although the dataset captured the majority of U.S. broiler chicken production, the effort only targeted the major companies. No attempts were made to determine the characteristics of broiler chicken production companies not included in this study. Third, granularity in the data varied by company, route of administration, and year.

Among the participating companies, there were several diseases for which antimicrobials were used frequently. First, diseases linked to *Clostridium spp*. remain an important cause of morbidity and mortality in broiler production. Necrotic enteritis, caused principally by *Clostridium perfringens* (19), ranked second among the disease-related issues that broiler production veterinarians faced in 2019 and has held that position for multiple years (20, 21). Gangrenous dermatitis, also known as clostridial dermatitis, is primarily caused by *Clostridium septicum* but also by *Clostridium perfringens* (22) and ranked fifth among the disease-related issues that broiler production veterinarians faced in 2019 (20). In the 2019 report from the United States Animal Health Association, broiler veterinarians reported that restricted-use antibiotic programs were a major challenge in production due to the inability to manage some of the most important diseases of broiler chickens, including diseases caused primarily or secondarily by coccidia (20).

Other important diseases of broilers are colibacillosis, which broadly refers to any localized or systemic infection caused entirely or partly by avian pathogenic *Escherichia coli* (APEC) (23), and respiratory diseases, which can also be caused by *E. coli*. Conditions such as septicemia, peritonitis, salpingitis, omphalitis/yolk sac infection, enteritis and others are all included in the category of colibacillosis; in this study, the respiratory disease category included airsacculitis. Hatchery antimicrobials are used primarily to prevent disease and reduce mortality associated with *E. coli*, such as omphalitis (yolk sac infection), but as stated previously, there was no reported hatchery AMU in 2020 or 2021.

The main diseases that necessitated treatment in the U.S. broiler dataset were similar to the diseases experienced in other countries. For example, in a study conducted by the EFFORT consortium in Europe that studied 181 broiler flocks across 9 European countries (24), the most commonly treated diseases across the countries were intestinal disorders, colibacillosis, omphalitis (which we grouped with colibacillosis), and respiratory disease. Most of the treatments in the European study were in the first week of life, with a fairly steady incidence of treatments at 15 through 37 days of age.

Although this study demonstrates that there were substantial reductions in AMU over the 2013–2021 period in U.S. broiler production, reduction of the total amounts of antimicrobial used should not be the primary goal of AMS programs. It should never be expected that total AMU will always decrease, as biological systems are inherently dynamic and the needs for antimicrobial therapy are always changing. As stated in the 2017 DANMAP report, “a few disease outbreaks in some farms can markedly affect and cause considerable fluctuations in the national statistics on antimicrobial usage. This was the case in late 2014 and throughout 2015” (25). The 2020 DANMAP report stated that AMU in poultry increased substantially from 2019 to 2020 due to increases in infections requiring treatment (26). Specifically, the report states that there were several *E. coli* outbreaks in older birds as well as overall increases in respiratory disease and enteritis that resulted in increased usage of tetracyclines and macrolides, respectively (26). According to the UK report for 2021, the use of fluoroquinolones in broilers increased from 2020 to 2021, although no explanation for the increase is provided (27). The incidence of disease dictates antimicrobial use patterns, assuming that antimicrobials are being used when necessary. Increases in annual antimicrobial use or sales should not necessarily be viewed as indicative of poor AMS, and conversely, decreases in antimicrobial use or sales should not alone be viewed as indicative of improved stewardship. The context for these changes is needed in order to understand the underlying reasons for these fluctuations.

Very few countries are currently collecting on-farm AMU data that are representative of national herds or flocks, such as this effort in the broiler chicken industry of the U.S. A recent paper characterized national systems for collecting on-farm data (4), but the effort we have developed in U.S. poultry was not included. Most of the efforts discussed in that paper were of limited sampling. For example, in Canada, a cross-sectional examination of farms is done each year, and the system collected data on 147 total broiler flocks nationally in 2019, representing approximately 3,474,669 total broilers (28). The approach this program in Canada uses is to select sentinel flocks from several provinces, with the number flocks proportional to the number of quota-holding producers; however, it is unclear how representative this sampling is to annual national production or antimicrobial use. A study published by the EFFORT consortium focused on antimicrobial use in 181 broiler flocks across nine European countries (24). As stated by the authors, the selection of 20 farms per country is not intended to be representative of broiler production in the country. Further, antimicrobial classes such as fluoroquinolones and polymyxins were used in multiple countries in this study; both of these classes are illegal for use in U.S. poultry, with fluoroquinolone use in U.S. poultry being eliminated since September 2005 (29). This disparity in antimicrobial approvals internationally highlights the challenges with comparing AMU across countries. Even for a national system such as DANMAP in Denmark (30), the data in the report are primarily antimicrobial sales; the usage data for poultry are not divided by species and provide no information regarding indication for use or any specific antimicrobial regimen data. In a study of broilers in Germany conducted from 2013 to 2018, 2,546 commercial broiler chicken flocks originating from 37 farms were followed over time (31). In total, approximately 78 million broiler chickens were surveyed, representing about 2.1% of the total number of broiler chickens slaughtered in Germany between 2013 and 2018. The study does many calculations of the AMU data but does not include information about the diseases that necessitated the reported AMU.

Globally, antimicrobial sales data continue to be the most commonly collected information at the national level, in part due to the ease of gathering these data when compared to collecting on-farm antimicrobial usage data. Sales data lack context and do not inform AMS and should not be used for setting antimicrobial reduction targets. AMS is not about reducing sales but rather ensuring that antimicrobials are used appropriately, which requires more information than simply amounts sold for use.

We have established a nationally representative on-farm system for collecting AMU data from commercial broiler chicken production in the U.S. In the second phase of this project, we were able to collect granular flock-level data for the water-soluble prescriptions. The first phase of this project focused primarily on antimicrobial totals used in broiler production over time (6), but this second phase now has detailed information about diseases targeted by specific antimicrobial administrations, age of birds at time of administration, and duration of treatment. This additional information can help the industry focus on those diseases that are necessitating the majority of antimicrobial use, especially the medically important antimicrobials. For the 2018–2021 dataset, the clostridial diseases NE and GD continued to be important reasons for antimicrobial use. These diseases deserve a continued focus on finding efficacious and cost-effective interventions. This project highlights a successful public-private partnership to enable collection and protection of sensitive flock-level data from an extremely large industry while releasing deidentified and aggregated information regarding the details of AMU on U.S. broiler chicken farms over time.

## Data availability statement

The original contributions presented in the study are included in the article/Supplementary material, further inquiries can be directed to the corresponding author.

## Author contributions

RS conceived of the project, recruited the companies, coordinated data acquisition, and wrote the manuscript drafts. RS, NS, and IR conducted the data analysis. NS and MA contributed to the paper content. NS, MA, and IR commented on manuscript drafts. All authors contributed to the article and approved the submitted version.
