# Supplementary material for: Antimicrobial usage in broiler chicken production in the United States, 2013–2021
Source: Front Vet Sci. 2023 Apr 17;10:1139908. doi: 10.3389/fvets.2023.1139908 (PMC10150104; doi:10.3389/fvets.2023.1139908)
Supplement: Supplementary file 1 [file Table_1.PDF]

Table S1. Antimicrobials that are Medically Important (MI) and that were used in broiler chicken production in the U.S. in 2021, categorized by route of administration. Antimicrobial drugs that were used within each class are shown as well as classification of importance per Appendix A of FDA's GFI #152 (U.S. Food and Drug Administration, 2003).

Route of Administration	Drug Class	Classification	Active Ingredient
**Injectable**			
	Aminoglycosides	Highly Important	Gentamicin
**Feed**			
	Diaminopyrimidines	Critically Important	Ormetoprim
	Streptogramins	Highly Important	Virginiamycin
	Sulfonamides	Critically Important	Sulfadimethoxine
	Tetracyclines	Highly Important	Chlortetracycline<br>Oxytetracycline
**Water**			
	Aminoglycosides	Highly Important	Neomycin<br>Spectinomycin
	Lincosamides	Highly Important	Lincomycin
	Macrolides	Critically Important	Tylosin
	Natural penicillins	Highly Important	Penicillin G
	Sulfonamides	Critically Important	Sulfadimethoxine<br>Sulfamerazine<br>Sulfamethazine<br>Sulfaquinoxaline
	Tetracyclines	Highly Important	Chlortetracycline<br>Oxytetracycline<br>Tetracycline

Table S2. Antimicrobials that are Not Medically Important (NMI) and that were used in broiler chicken production in the U.S. in 2021, categorized by route of administration. Antimicrobial drugs that were used within each class are shown.

Route of Administration	Drug Class	Active Ingredient
**Feed**		
	Glycolipids	Bambermycins
	Ionophores	Lasalocid
		Monensin
		Narasin
		Salinomycin
	Orthosomycins	Avilamycin
	Polypeptides	Bacitracin
**Water**		
	Polypeptides	Bacitracin

REFERENCES

U.S. Food and Drug Administration (2013). *Guidance for Industry #213: New Animal Drugs and New Animal Drug Combination Products Administered in or on Medicated Feed or Drinking Water of Food-Producing Animals: Recommendations for Drug Sponsors for Voluntarily Aligning Product Use Conditions with GFI #209*. Retrieved from https://www.fda.gov/regulatory-information/search-fda-guidance-documents/cvm-gfi-213-new-animal-drugs-and-new-animal-drug-combination-products-administered-or-medicated-feed. Last accessed November 22, 2022.
